# Anti-Dengue Virus Constituents from Formosan Zoanthid *Palythoa mutuki*

**DOI:** 10.3390/md14080151

**Published:** 2016-08-09

**Authors:** Jin-Ching Lee, Fang-Rong Chang, Shu-Rong Chen, Yu-Hsuan Wu, Hao-Chun Hu, Yang-Chang Wu, Anders Backlund, Yuan-Bin Cheng

**Affiliations:** 1 Graduate Institute of Natural Products, College of Pharmacy, Kaohsiung Medical University, Kaohsiung 807, Taiwan; jclee@kmu.edu.tw (J.-C.L.); aaronfrc@kmu.edu.tw (F.-R.C.); highshorter@hotmail.com (S.-R.C.); drjcount@livemail.tw (H.-C.H.); 2 Department of Biotechnology, College of Life Science, Kaohsiung Medical University, Kaohsiung 807, Taiwan; 3 Research Center for Natural Products and Drug Development, Kaohsiung Medical University, Kaohsiung 807, Taiwan; 4 Department of Marine Biotechnology and Resources, National Sun Yat-sen University, Kaohsiung 804, Taiwan; 5 Cancer Center, Kaohsiung Medical University Hospital, Kaohsiung 807, Taiwan; 6 Institute of Basic Medical Sciences, College of Medicine, National Cheng Kung University, Tainan 701, Taiwan; windywingsy@gmail.com; 7 Center of Infectious Disease and Signaling Research, College of Medicine, National Cheng Kung University, Tainan 701, Taiwan; 8 School of Pharmacy, College of Pharmacy, China Medical University, Taichung 404, Taiwan; yachwu@mail.cmu.edu.tw; 9 Chinese Medicine Research and Development Center, China Medical University Hospital, Taichung 404, Taiwan; 10 Center for Molecular Medicine, China Medical University Hospital, Taichung 404, Taiwan; 11 Research Center for Chinese Herbal Medicine, China Medical University, Taichung 404, Taiwan; 12 Division of Pharmacognosy, Department of Medicinal Chemistry, Uppsala University, BMC Box 574, S-751 23 Uppsala, Sweden; anders.backlund@fkog.uu.se; 13 Center for Infectious Disease and Cancer Research, Kaohsiung Medical University, Kaohsiung 807, Taiwan

**Keywords:** ecdysteroid, *Palythoa mutuki*, antiviral activity, ChemGPS–NP

## Abstract

A new marine ecdysteroid with an α-hydroxy group attaching at C-4 instead of attaching at C-2 and C-3, named palythone A (**1**), together with eight known compounds (**2**–**9**) were obtained from the ethanolic extract of the Formosan zoanthid *Palythoa mutuki*. The structures of those compounds were mainly determined by NMR spectroscopic data analyses. The absolute configuration of **1** was further confirmed by comparing experimental and calculated circular dichroism (CD) spectra. Anti-dengue virus 2 activity and cytotoxicity of five isolated compounds were evaluated using virus infectious system and [3-(4,5-dimethylthiazol-2-yl)-5-(3-carboxymethoxyphenyl)-2-(4-sulfophenyl)-2*H*-tetrazolium, inner salt (MTS) assays, respectively. As a result, peridinin (**9**) exhibited strong antiviral activity (IC_50_ = 4.50 ± 0.46 μg/mL), which is better than that of the positive control, 2′CMC. It is the first carotene-like substance possessing anti-dengue virus activity. In addition, the structural diversity and bioactivity of the isolates were compared by using a ChemGPS–NP computational analysis. The ChemGPS–NP data suggested natural products with anti-dengue virus activity locate closely in the chemical space.

## 1. Introduction

Zoanthid of the genus *Palythoa* (family Sphenopidae) is a kind of benthos commonly found in shallow waters. More than 90 species of this genus were identified in subtropical and tropical areas all over the world. This marine creature is characterized by absorbing sands or small sediments into polyp to reinforce their structure. Apart from the well-known poisonous compound, palytoxin [1], *Palythoa* zoanthids were also reported to produce various natural products, such as amino acids [2,3], steroids [4], ecdysteroids [5], prostanoids [6], and sulfonylated ceramides [7]. The natural products of zoanthids not only act as defensive substance against predators, but also exhibit diverse bioactivities for the development of new drugs. For example, a polyhydroxylated steroid isolated from *P. tuberculosa* selectively inhibited human breast cancer cells (MCF-7), which implied this compound might be a new anti-cancer therapeutic agent [8]. Recently, our research group has studied anti-dengue virus ecdysteroids from Formosan zoanthid *Zoanthus* spp. [9]. In our continuous screening for bioactive marine natural products, the ethanolic extract of *P. mutuki* showed strong anti-dengue virus activity. Because there is no medicine for dengue fever, the animal materials of *P. mutuki* were investigated for its bioactive ingredients. In this manuscript, the isolation, structural elucidation, antiviral activity, and ChemGPS–NP space mapping analysis of one new and eight known compounds from *P. mutuki* are described.

## 2. Results and Discussion

**Figure 1:**
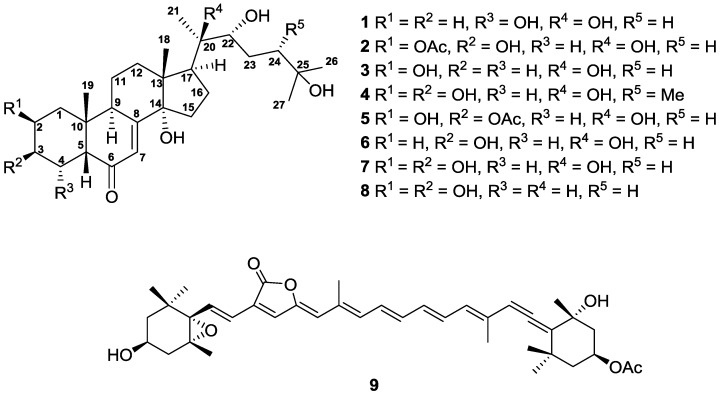
Structures of compounds **1**−**9**.

The animal materials of *P. mutuki* were collected in the northeast coast of Taiwan. The sample was extracted by ethanol and partitioned between 75% methanol and hexanes to give two different organic extracts. Repeated column chromatography of the 75% methanol extract yielded one new compound, palythone A (**1**), and eight known compounds, 20-hydroxyecdysone 2-acetate (**2**) [10], 3-deoxy-20-hydroxyecdysone (**3**) [11], 24-*epi*-makisterone A (**4**) [12], 20-hydroxyecdysone 3-acetate (**5**) [10], 2-deoxyecdysterone (**6**) [13], 20-hydroxyecdysone (**7**) [14], α-ecdysone (**8**) [15], and peridinin (**9**) [16]. The structures of all isolates were determined according to their spectroscopic data and are shown in Figure 1.

Palythone A (**1**), ${\left[\alpha \right]}_{D}^{26}$ −11 (*c* 0.05, MeOH), was obtained as a white amorphous powder. From its HRESIMS data (*m*/*z* 487.3028 [M + Na]^+^) and ^13^C NMR spectrum, a molecular formula of C_27_H_44_O_6_ and six degrees of unsaturation were established. The infrared (IR) spectrum of **1** revealed the presence of hydroxy (3372 cm^−1^), ketone (1650 cm^−1^), and C-O (1089 cm^−1^) groups. The ^1^H and ^13^C NMR data of **1** are summarized in Table 1. The ^1^H data revealed the presences of five methyl singlets (δ_H_ 1.01, 1.25, 1.40, 1.40, and 1.61), one olefinic methine doublet (δ_H_ 6.25, *J* = 2.6 Hz), two oxymethines (δ 3.85 and 3.90), and two aliphatic methines (δ 3.03 and 3.72). In the ^13^C NMR and distortionless enhancement by polarization transfer (DEPT) spectra of **1**, twenty-seven carbon signals including one carbonyl (δ 202.0), one olefinic methine (δ 121.4), one olefinic quaternary carbon (δ 166.3), two oxymethines (δ 69.2 and 77.6), three aliphatic methines (δ 34.0, 50.1, and 57.3), two aliphatic quaternary carbons (δ 36.8 and 48.2), three oxygen-bearing quaternary carbons (δ 69.6, 76.9, and 84.2), nine aliphatic methylenes (δ 20.9, 21.5, 27.5, 31.7, 31.7, 32.0, 34.3. 35.7, and 42.7), and five methyls (δ 17.9, 21.7, 23.9, 30.0, and 30.1) were observed. The above data and the UV maximum absorption at 245 nm implied that **1** should belong to ecdysteroid [13].

**Figure 2:**
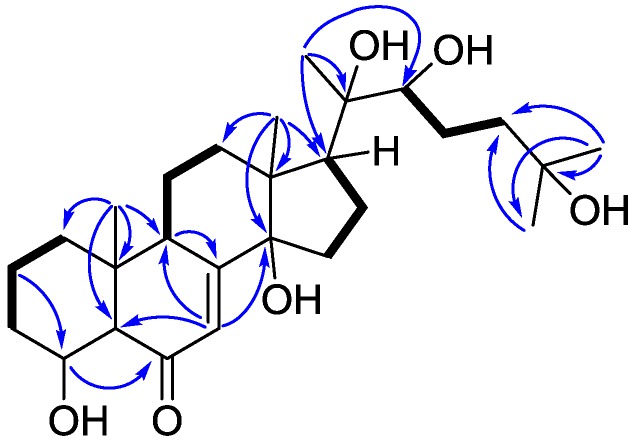
COSY (bold bond) and selected HMBC (arrow) correlations of **1**.

Three partial structures of H_2_-1 (δ 1.88 and 1.11)/H_2_-2 (δ_H_ 2.08 and 1.81)/H_2_-3 (δ 2.17 and 1.92), H-9 (δ 3.72)/H_2_-11 (δ 1.80 and 1.70)/H_2_-12 (δ 2.60 and 2.06), and H_2_-15 (δ 2.00 and 1.85)/H_2_-16 (δ 2.48 and 2.10)/H-17 (δ 3.03) were revealed by the COSY spectrum (Figure 2). In the HMBC spectrum of **1**, correlations of Me-19 (δ 1.01)/C-1 (δ 34.3), C-5 (δ 57.3), C-9 (δ 34.0), C-10 (δ 36.8) and Me-18 (δ 1.25)/C-12 (δ 32.0), C-13 (δ 48.2), C-14 (δ 84.2), C-17 (δ 50.1) can be used to link the above mentioned partial structures. Moreover, the presence of the characteristic 7-en-6-one tetracyclic ring system in **1** was confirmed by the HMBC correlations of H_2_-2/C-4 (δ 69.2), H-4 (δ 3.85)/C-6 (δ 202.0), H-9/C-8 (δ 166.3), and H-7 (δ 6.25)/C-5, C-9, C-14. The aliphatic side chain from C-20 to C-27 was validated by the HMBC correlations of Me-21 (δ 1.61)/C-20 (δ 76.9), C-22 (δ 77.6), Me-26 (δ 1.40)/C-24 (δ 42.7), C-25 (δ_C_ 69.6), C-27 (δ_C_ 30.0), along with the COSY correlations of H-22 (δ 3.90)/H_2_-23 (δ 2.14 and 1.86)/H_2_-24 (δ 2.28 and 1.85). Finally, the HMBC correlations from Me-21 to C-17 proved the side chain was situated at C-17. Thus, the planar structure of **1** was established.

**Figure 3:**
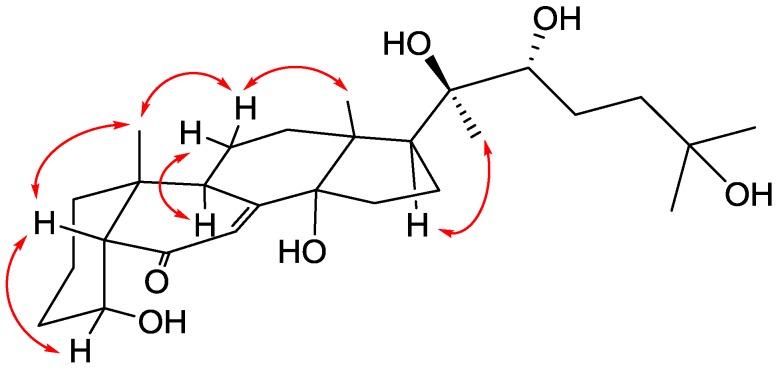
Key NOESY (left right arrow) correlations of **1**.

**Figure 4:**
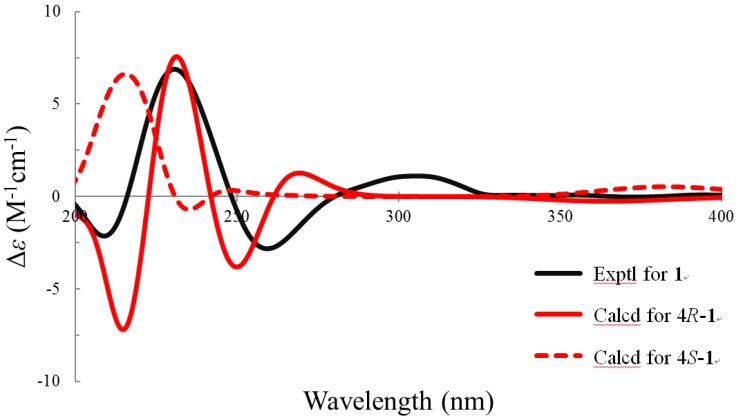
Calculated and experimental CD spectra of **1**.

The stereochemistry of **1** was determined by the NOESY correlations (Figure 3) and confirmed by the CD experiments. The *cis*-fused geometry of rings A and B were determined by means of the NOESY correlations between Me-19 and H-5 (δ 2.32). The hydroxy group attached at C-4 was placed on the α-face according to the presence of the NOESY correlation between H-4 and H-5 and the absence of NOESY correlation between H-4 and H-9. To confirm the absolute configuration of C-4, the ECD experiment was carried out. The experimental CD spectrum of **1** demonstrated two positive bands (231 and 306 nm) and two negative bands (209 and 259 nm), which resembled the calculated ECD spectra of 4*R*-**1** (Figure 4). Thus, the absolute configuration of C-4 was defined to be *R*. The NOESY cross-peaks of Me-19/H-11β (δ 1.70)/Me-18 revealed that these protons are on the β-face of the molecule. On the other hand, the presence of NOESY correlations of H-9/H-11α (δ 1.80) and H-17/Me-21 indicated that these protons locate on the α-face. The *R* configurations of both C-20 and C-22 were determined by comparing proton chemical shifts of **1** with related ecdysteroids [17]. Therefore, structure of **1** was determined unambiguously.

Due to the anti-dengue virus activities of some ecdysteroids (**5**–**8**) that were revealed previously [9], the other five compounds (**1**–**4**, and **9**) were selected to evaluate their anti-dengue virus 2 (DENV-2) activities and cytotoxicity. As a result, palythone A (**1**) and 24-*epi*-makisterone (**4**) demonstrated weak anti-DENV-2 activities (Table 2). Unexpectedly, compound **9** exhibited the most potent antiviral activity with an EC_50_ value of 4.50 ± 0.46 μM. This activity is superior to that of the positive control, 2′CMC, which was previously reported as a specific anti-DENV agent in vitro and in vivo [18].

In addition, the ability to suppress virus production of peridinin (**9**) was measured by a TCID_50_ assay. The result showed that **9** at dose of 10 μM effectively decreased the viral titer by 2 ± 0.6 log_10_ as compared to mock-treatment. Peridinin (**9**) was also tested for other sero-types of DENV, and the results are shown in Table 3. The results indicated that it can inhibit all sero-types of DENV.

Moreover, the anti-DENV protease activity of peridinin (**9**) was characterized by using NS3 protease reporter-based assay. The results showed this compound exhibited inhibitory effect on DENV protease activity with an EC_50_ value of 8.50 ± 0.41 μM. To date, no approved agents for treating DENV infection is available, thus there is an urgent need to develop potential anti-DENV agents. Currently, some natural products have been identified to exhibit anti-DENV activity. For example, Zandi et al. have reported that the *Scutellaria baicalensis* extract and quercetin exhibited anti-DENV activity with IC_50_ value of 93.66 μg/mL (SI = 9.74) and 35.7 μg/mL (SI = 7.07), respectively [19,20]. In addition, Brandão et al. have identified the anti-DENV activity of *Arrabidaea pulchra* extract with an IC_50_ value of 46.8 ± 1.6 μg/mL (SI = 2.7) [21]. Those natural products exhibited lower anti-DENV activity and SI value than that of peridinin (**9**). To determine the applicability of peridinin (**9**), the in vivo tests will be performed in the future. Furthermore, future studies about computer-aided development of pharmacophoric models should be performed to optimize the antiviral activity of peridinin (**9**). Since there is no antiviral drug against DENV infection, our study identifies a potential lead compound for novel anti-DENV agent development.

**Figure 5:**
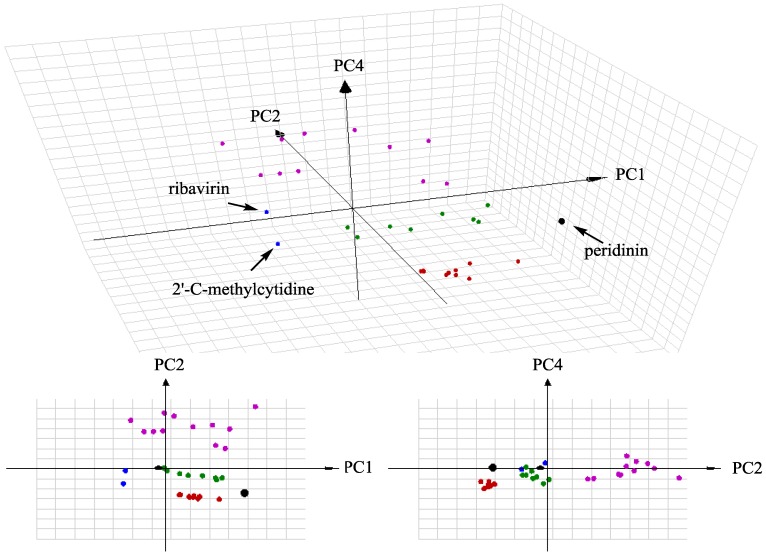
ChemGPS–NP space coordinates of peridinin (**black**), ecdysteroids (**red**), limonoids (**green**), positive control (**blue**), and non-peptidic anti-dengue virus compounds (**purple**).

On the basis of the chemical space concept, a computational high-throughput screening method named the ChemGPS–NP was advanced by Josefin et al. [22,23]. ChemGPS–NP is a principal component analysis (PCA) based coordinate system with eight dimensions, which mainly concerns the size, shape, and polarizability (PC1), aromatic- and conjugation-related properties (PC2), lipophilicity, polarity, and H-bond capacity (PC3), and flexibility (PC4) of natural products. There is a theory which states that compounds with similar physico-chemical properties could possess comparable bioactivities and mechanisms [24]. Therefore, the molecular diversity of the isolates and three anti-dengue virus datasets was analyzed by the ChemGPS–NP map of chemical space. The first dataset consisted of the seventeen anti-dengue virus ecdysteroids isolated from zoanthids; the second dataset contained eight anti-dengue virus limonoids separated from *Swietenia macrophylla* [25]; and the third dataset composed of twelve non-peptidic anti-dengue virus compounds [26,27]. The score plot of three descriptors (PC1, PC2, and PC4) revealing that peridinin (**9**), limonoids, and ecdysteroids situated in the same quadrant (Figure 5), and, meanwhile, peridinin and limonoids occupied the same quadrant while the descriptors changed to PC1, PC2, and PC3. Furthermore, two positive control compounds (ribavirin and 2′CMC) and twelve non-peptidic anti-dengue virus compounds placed in different quadrants away from all natural products. Our findings suggested natural products located in the specific chemical space might be possible new anti-dengue virus agents.

## 3. Materials and Methods

### 3.1. General Experimental Procedures

Optical rotation was measured on a JASCO P-1020 digital polarimeter (Tokyo, Japan). UV data were recorded on a JASCO V-530 UV/VIS Spectrophotometer (Tokyo, Japan). CD spectrum was acquired on a JASCO J-815 CD spectrometer (Tokyo, Japan). High-resolution ESIMS data were obtained on a Bruker APEX II spectrometer (Billerica, MA, USA). IR spectrum was measured on a Perkin Elmer system 2000 FT-IR spectrophotometer (Waltham, MA, USA). NMR spectra were obtained by Varian 600 MHz NMR (San Carlos, CA, USA). Merck silica gel 60 (Billerica, MA, USA) and Sephadex LH-20 (Stockholm, Sweden) were used for column chromatography. The instrumentation for HPLC was composed of a Shimadzu LC-10AD pump (Kyoto, Japan) and a Shimadzu SPD-M10A PDA detector (Kyoto, Japan).

### 3.2. Animal Material

Specimens of *Palythoa mutuki* were collected in Keelung City, Taiwan, in August 2015. The research samples were identified by its 16S rDNA gene sequence. A voucher specimen (no. KMU-MrPm) was deposited in the Graduate Institute of Natural Products, College of Pharmacy, Kaohsiung Medical University.

### 3.3. Species Identification

Samples were preserved in 75% ethanol at ambient temperature. DNA was extracted by a DNeasy Plant Mini Kit (Qiagen #68163, Venlo, The Netherlands). Two sets of primers 16Santa1a: 5′-GCCATGAGTATAGACGCACA-3′/16SbmoH: 5′-CGAACAGCCAACCCT TGG-3′ and HCO2198:5′-TAAACTTCAGGGTGACCAAAAAATCA-3′/LCO1490: 5′-GGTCAACAAATCATAAAGATA TTGG-3′ were chosen to amplify the mitochondrial 16S (mt 16S), respectively. PCR amplifications were worked using FlexCycler^2^ (analytik jena) (Jena, Germany) with the latter conditions: 94 °C (1 min), 40 cycles of 98 °C (10 s), 52 °C (1 min), and 68 °C (1 min), with the last extension at 68 °C (5 min). The purified PCR products were analyzed by Genomics BioSci & Tech. (New Taipei City, Taiwan) for sequencing services. The mt 16S rDNA gene sequence were compared with NCBI database. Consequently, the research sample shared 100% sequence identity with *Palythoa mutuki* (GenBank: DQ997847.1).

### 3.4. Extraction and Isolation

The animal material was extracted by ethanol three times and partitioned between ethyl acetate and water to give an ethyl acetate-soluble extract. This extract was further partitioned between hexanes and 75% methanol for separating low polar compounds. The 75% methanol-soluble extract (11.9 g) was subjected to a Sephadex LH-20 column (Stockholm, Sweden) eluted with methanol to give four fractions (L1–L4). Fraction L2 (1.7 g) was separated by a Si gel column eluted with methylene chloride and methanol (12:1 to 0:1) to furnish fourteen fractions (L2S1–L2S14). Fraction L2S7 (119.3 mg) was purified by HPLC (Luna phenyl-hexyl, 10 mm × 250 mm, flow rate, 2.0 mL/min, 23% acetonitrile) to afford compounds 5 (33.8 mg) and 2 (49.5 mg). Fractions L2S8 (26.9 mg) was isolated by HPLC (Luna phenyl-hexyl, 10 mm × 250 mm, flow rate = 2.0 mL/min, 23% acetonitrile) to yield compounds 1 (0.6 mg), 3 (0.8 mg) and 6 (4.1 mg). Fractions L2S9 (83.1 mg) was subjected to HPLC (Luna phenyl-hexyl, 10 mm × 250 mm, flow rate = 2.0 mL/min, 20% acetonitrile) to give compound 4 (7.6 mg), 7 (0.4 mg), and 8 (1.0 mg). Fraction L3 (1.1 g) was separated by a Si gel column eluted with hexane, ethyl acetate, and methanol (15:1:0 to 0:0:1) to furnish ten fractions (L3S1–L3S10). Fractions L3S4 (90.6 mg) was isolated by HPLC (Luna C_18_, 10 mm × 250 mm, flow rate = 2.0 mL/min, 85% methanol) to obtain compound 9 (1.5 mg).

Palythone A (1): White amorphous powder; ${\left[\alpha \right]}_{D}^{26}$ −11 (*c* 0.05, MeOH); UV (MeOH) *λ*_max_ (log *ε*) 245 (3.50) nm; CD (MeOH) *λ*_max_ (Δ*ε*): 209 (−2.14), 231 (+6.88), 259 (−2.82), 306 (+1.11) nm; IR (neat) ν_max_: 3372, 2933, 1650, 1559, 1417, 1234, 1089, 889 cm^−1^; ^1^H NMR and ^13^C NMR data, see Table 1 and Supplementary Materials; HRESIMS *m*/*z* 487.3028 [M + Na]^+^ (calcd. for C_27_H_44_O_6_Na, 487.3030).

### 3.5. ECD Calculations

The minima energies of 4*R*-1 and 4*S*-1 were calculated by ChemBio3D (ver. 14.0, Perkin Elmer, Waltham, MA, USA) and the structure of 4*R*-1 and 4*S*-1 were saved as tinker MM2 input files. These data were imported into the Gaussian 09 for density functional theory (DFT) at the B3LYP/6-31G(d) level in the gas phase to obtain the restricted conformations. The energies, rotational strengths, and oscillator strengths of the 20 weakest conformers were optimized using the time-dependent density functional theory (TDDFT) methodology at the B3LYP/6-311++G(d,p) level. The final ECD files were converted to txt files by GaussSum 2.2.5 (Gaussian Inc., Wallingford, CT, USA) with a bandwidth σ of 0.5 eV. The ECD and CD curves were plotted by Excel.

### 3.6. Anti-DENV Activity Assay

Huh-7 cells were cultured in Dulbecco’s modified Eagle’s medium (DMEM) containing 10% fetal bovine serum, 1% non-essential amino acids, and 1% antibiotic-antimycotic in a 5% CO_2_ at 37 °C. Huh-7 cells were seeded at 24-well plate at density 5 × 10^4^ cells/well and infected by DENV infection at a multiplicity of infection (MOI) of 0.2 followed by test compounds treatment 2 h post infection. Total cellular RNA were harvested at 72 h post-infection, and DENV RNA level was analyzed using quantitative real-time reverse-transcription polymerase chain reaction (qRT-PCR) with specific primers against DENV NS5 gene. DENV RNA level was normalized by cellular glyceraldehydes-3-phosphate dehydrogenase (gapdh) mRNA level. 2′-C-methylcytidine (2′CMC) served as positive control. DENV of different serotypes (DENV-1: DN8700828; DENV-2: DN454009A; DENV-3: DN8700829A; DENV-4: S9201818) were obtained from the Centers for Disease Control, Department of Health, Taipei, Taiwan.

### 3.7. Evaluation of Anti-DENV RdRp and Protease Activity

The DENV RdRp activity reporter system was used to determine the anti-DENV RdRp activity as described before [18]. The NS3 protease reporter-based assay was used to determine the anti-DENV protease activity. The Huh-7 cells were transfected with DENV NS2B/NS3 protease reporter vector pEG(∆4B/5)NLuc carrying a specific DENV protease cleavage peptide sequences and the DENV protease expression vector. Subsequently, the cells were treated with compound 9 for 3 days. The supernatant was collected to analyze the nano luciferase (NLuc) activity by Nano-Glo^®^ Luciferase Assay System following the manufacturer’s instructions (Promega, Madison, WI, USA).

### 3.8. Cytotoxicity Assay

Huh-7 cells were seeded onto 96-well plate at a density of 5 × 10^3^ cells per well, followed by compound treatment for 72 h. The cell viability was determined using a standard MTS assay (CellTiter 96^®^ Aqueous One Solution Cell Proliferation assay system, Promega, Madison, WI, USA) according to the manufacturer’s instructions.

### 3.9. ChemGPS–NP Analysis

Anti-dengue virus compounds were classified into three datasets (17 ecdysteroids, 8 limonoids, and 12 non-peptidic compounds), 2 positive control compounds, and peridinin. The structures of all compounds were converted to line notations (SMILES). The SMILES data were submitted to ChemGPS–NP_web_ (http://chemgps.bmc.uu.se) for mapping chemical space. The results were plotted with 3D grapher 1.21 (RomanLab software, Vancouver, BC, Canada).

## 4. Conclusions

In our continuous investigation on discovering anti-dengue virus natural products, the Formosan zoanthid *Palythoa mutuki* has resulted in the identification of one new ecdysteroid (**1**) and eight known compounds (**2**–**9**). The potent anti-dengue virus activity of peridinin (**9**), a common secondary metabolite in marine invertebrates and dinoflagellates was discovered for the first time. Our findings suggest carotenoid-like substance might possess anti-dengue virus activity. Zoanthids have become one of the good resources for antiviral natural product development.

## Figures and Tables

**Table 1:** ^1^H and ^13^C NMR Data of **1** in C_5_D_5_N *^a^*.

Position	δ_H_, Mult (*J* in Hz)	δ_C_, Type	HMBC (^1^H–^13^C)
1	1.88, m	34.3, CH_2_	
	1.11, m		
2	2.08, m	35.7, CH_2_	4, 10
	1.81, m		
3	2.17, m	31.7, CH_2_	
	1.92, dd (9.4, 2.7)		
4	3.85, m	69.2, CH	6
5	2.32, dd (13.3, 4.0)	57.3, CH	6
6		202.0, C	
7	6.25, d (2.8)	121.4, CH	5, 9, 14
8		166.3, C	
9	3.72, ddd (12.8, 6.0, 2.8)	34.0, CH	8, 11
10		36.8, C	
11α	1.80, m	20.9, CH_2_	
11β	1.70, m		
12	2.60, td (12.8, 4.7)	32.0, CH_2_	9, 11, 13, 14, 18
	2.06, m		11, 13, 18
13		48.2, C	
14		84.2, C	
15	2.00, m	31.7, CH_2_	14
	1.85, m		
16	2.48, m	21.5, CH_2_	
	2.10, m		
17	3.03, t (9.5)	50.1, CH	
18	1.25, s	17.9, CH_3_	12, 13, 14, 17
19	1.01, s	23.9, CH_3_	1, 5, 9, 10
20		76.9, C	
21	1.61, s	21.7, CH_3_	17, 20, 22
22	3.90, dd (9.8, 3.3)	77.6, CH	
23	2.14, m	27.5, CH_2_	
	1.86, m		
24	2.28, dd (11.8, 3.4)	42.7, CH_2_	
	1.85, m		25, 26, 27
25		69.6, C	
26	1.40, s	30.1, CH_3_	24, 25, 27
27	1.40, s	30.0, CH_3_	24, 25, 26

*^a^* ^1^H and ^13^C NMR data (δ) were measured at 600 and 150 MHz, respectively; Chemical shifts are in ppm.

**Table 2:** Anti-DENV-2 activity of selected compounds.

Compound	EC_50_ (μM) *^a^*	CC_50_ (μM) *^b^*	SI *^c^*
**1**	54.01 ± 2.17	NT *^d^*	–
**2**	>100	>200	–
**3**	>100	>200	–
**4**	46.45 ± 0.59	NT *^d^*	–
**9**	4.50 ± 0.46	132.55 ± 3.21	>29.5
2′CMC *^e^*	13.23 ± 0.07	94.8 ± 4.15	>7.2

*^a^* Half maximal effective concentration; *^b^* Half maximal cytotoxicity concentration; *^c^* Selectivity index (SI), CC_50_/EC_50_; *^d^* Not tested; *^e^* 2′-C-methylcytidine, positive control.

**Table 3:** Anti-DENV-1-4 activity of peridinin (**9**).

EC_50_ (μM) *^a^*			
DENV-1	DENV-2	DENV-3	DENV-4
7.62 ± 0.17	4.50 ± 0.46	5.84 ± 0.19	6.51 ± 0.30

*^a^* Half maximal effective concentration.

## Supplementary Materials

The following are available online at www.mdpi.com/1660-3397/14/8/151/s1.
