# Supplementary material for: Anti-Dengue Virus Constituents from Formosan Zoanthid Palythoa mutuki
Source: Mar Drugs. 2016 Aug 9;14(8):151. doi: 10.3390/md14080151 (PMC4999912; doi:10.3390/md14080151)
Supplement: Supplementary file 1 [file marinedrugs-14-00151-s001.pdf]

Supplementary Materials: Anti-Dengue Virus Constituents from Formosan Zoanthid *Palythoa mutuki*

Jin-Ching Lee, Fang-Rong Chang, Shu-Rong Chen, Yu-Hsuan Wu, Hao-Chun Hu, Yang-Chang Wu, Anders Backlund and Yuan-Bin Cheng

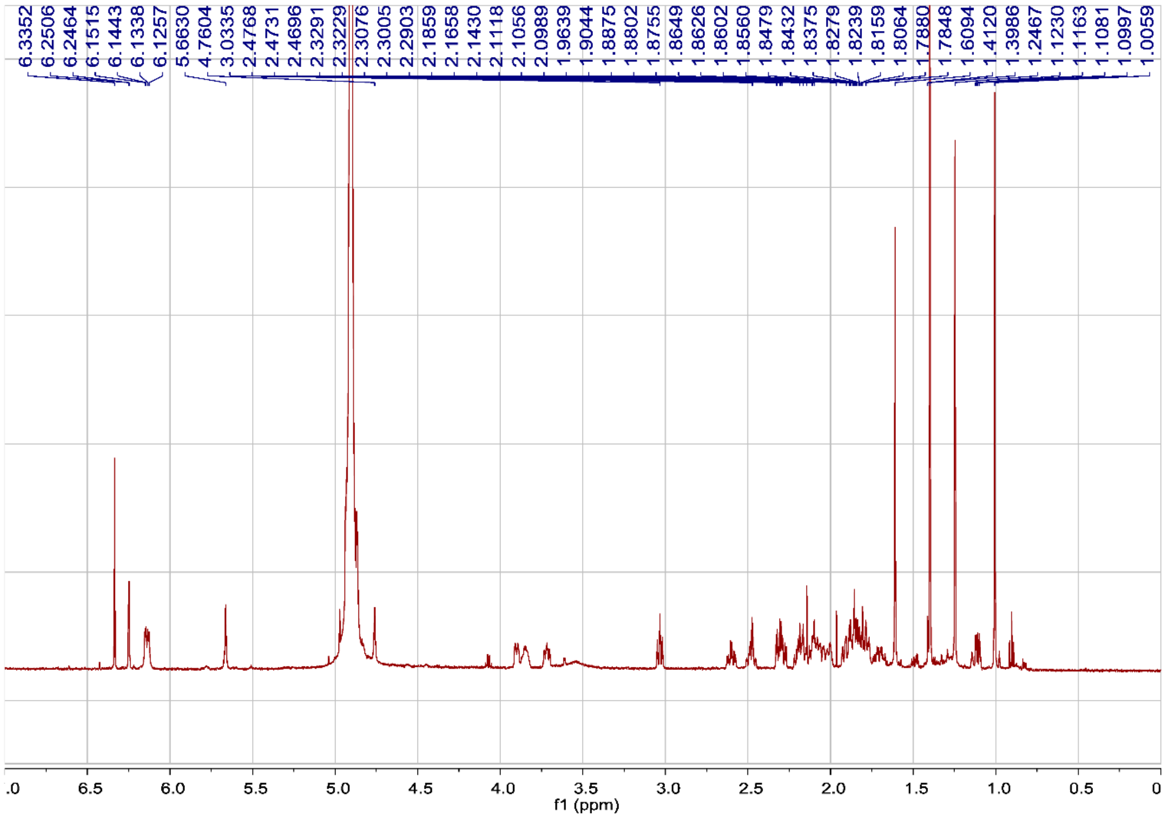

Figure S1. ^{1}H NMR spectrum (600 MHz, pyridine-d_5) of palythone A (**1**).

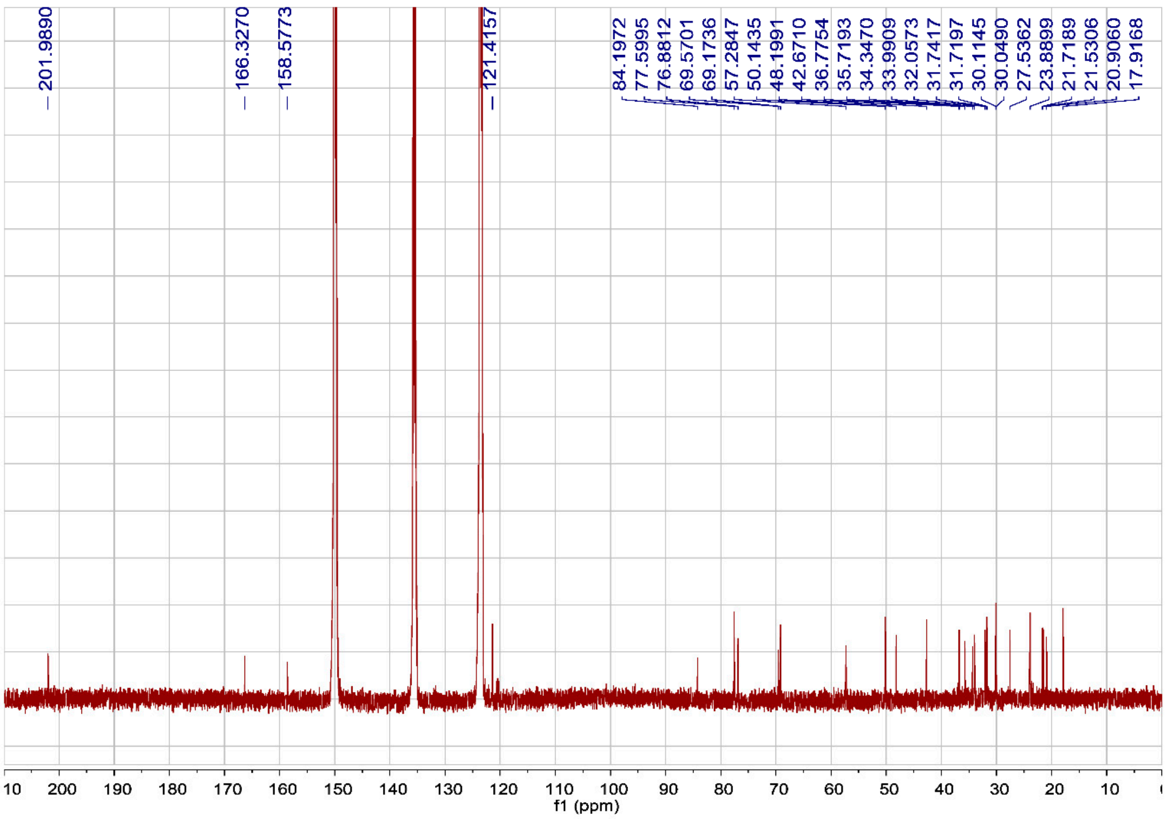

Figure S2. ^{13}C NMR spectrum (150 MHz, pyridine-d_5) of palythone A (**1**).

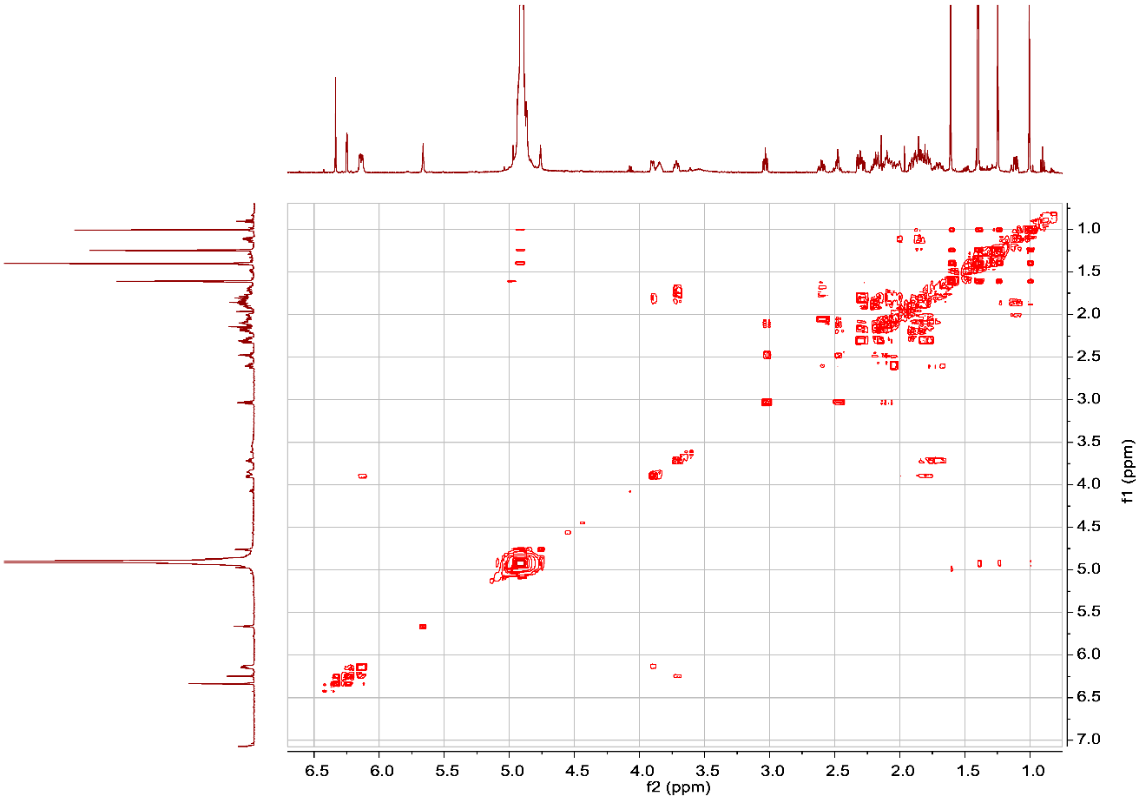

Figure S3. COSY spectrum (600 MHz, pyridine-d_5) of palythone A (**1**).

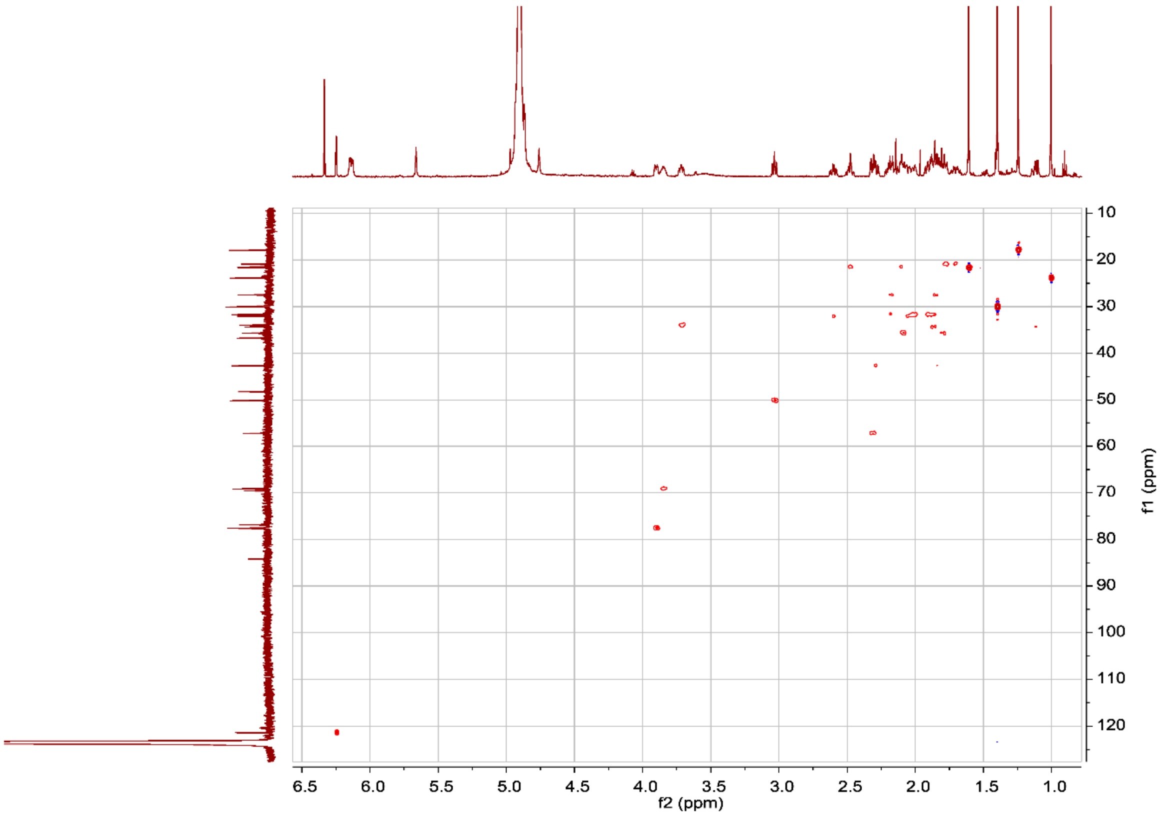

Figure S4. HSQC spectrum (600 MHz, pyridine-*d*5) of palythone A (**1**).

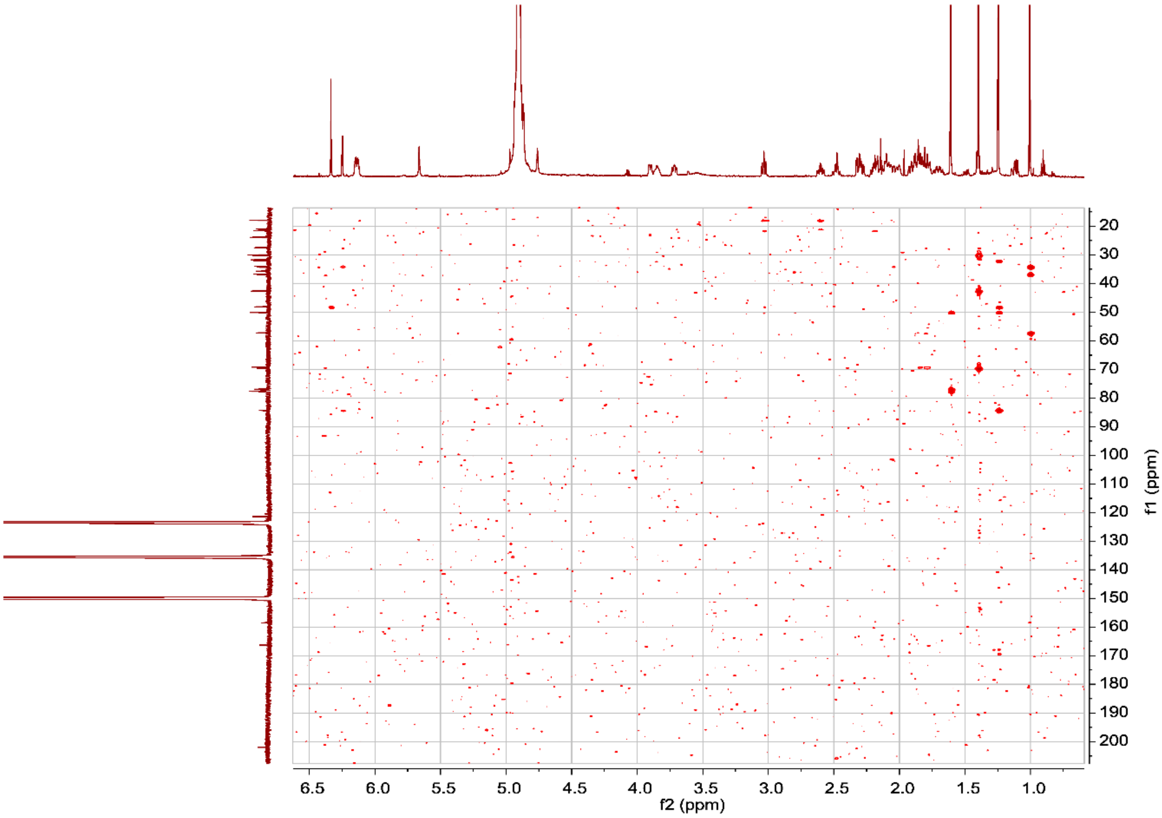

Figure S5. HMBC spectrum (600 MHz, pyridine-*d*5) of palythone A (**1**).

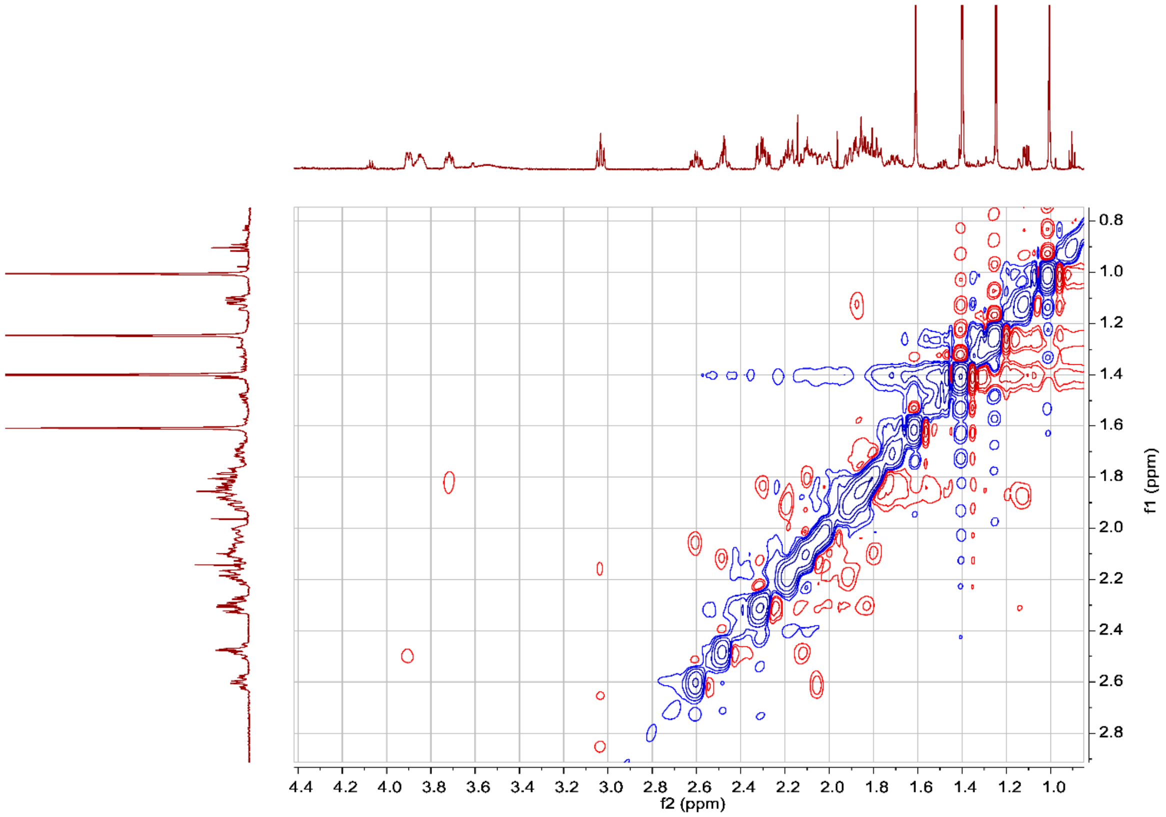

Figure S6. NOESY spectrum (600 MHz, pyridine-*d*5) of palythone A (**1**).

Dataset A: 17 ecdysteroids

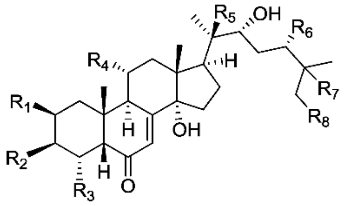

1 $R_1 = R_2$ = OH, R_3 = H, $R_4 = R_5$ = OH, R_6 = H, R_7 = OAc, R_8 = H
2 $R_1 = R_2$ = H, R_3 = OH, R_4 = H, R_5 = OH, R_6 = H, R_7 = OH, R_8 = H
3 $R_1 = R_2$ = OH, $R_3 = R_4$ = H, R_5 = OH, R_6 = H, R_7 = OAc, R_8 = H
4 $R_1 = R_2$ = OH, R_3 = H, $R_4 = R_5$ = OH, $R_6 = R_7 = R_8$ = H
5 R_1 = OH, R_2 = OAc, $R_3 = R_4$ = H, R_5 = OH, R_6 = H, R_7 = OH, R_8 = H
6 R_1 = OAc, R_2 = OH, $R_3 = R_4$ = H, R_5 = OH, R_6 = H, R_7 = OH, R_8 = H
7 $R_1 = R_2$ = OH, $R_3 = R_4$ = H, R_5 = OH, R_6 = H, R_7 = OH, R_8 = H
8 $R_1 = R_2$ = OH, $R_3 = R_4 = R_5 = R_6$ = H, R_7 = OH, R_8 = H
9 R_1 = H, R_2 = OH, $R_3 = R_4$ = H, R_5 = OH, R_6 = H, R_7 = OH, R_8 = H
10 R_1 = OH, $R_2 = R_3 = R_4$ = H, R_5 = OH, R_6 = H, R_7 = OH, R_8 = H
11 $R_1 = R_2$ = OH, $R_3 = R_4$ = H, R_5 = OH, $R_6 = R_7 = R_8$ = H
12, **13** $R_1 = R_2$ = OH, $R_3 = R_4$ = H, R_5 = OH, $R_6 = R_7$ = H, R_8 = OH
14 $R_1 = R_2$ = OH, $R_3 = R_4$ = H, $R_5 = R_6$ = OH, $R_7 = R_8$ = H
15 $R_1 = R_2$ = OH, R_3 = H, $R_4 = R_5$ = OH, R_6 = H, R_7 = OH, R_8 = H

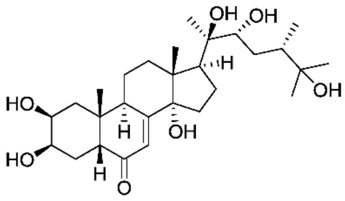

16

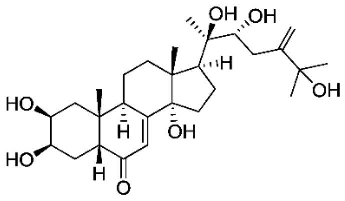

17

Figure S7. *Cont.*

Dataset B: 8 limonoids

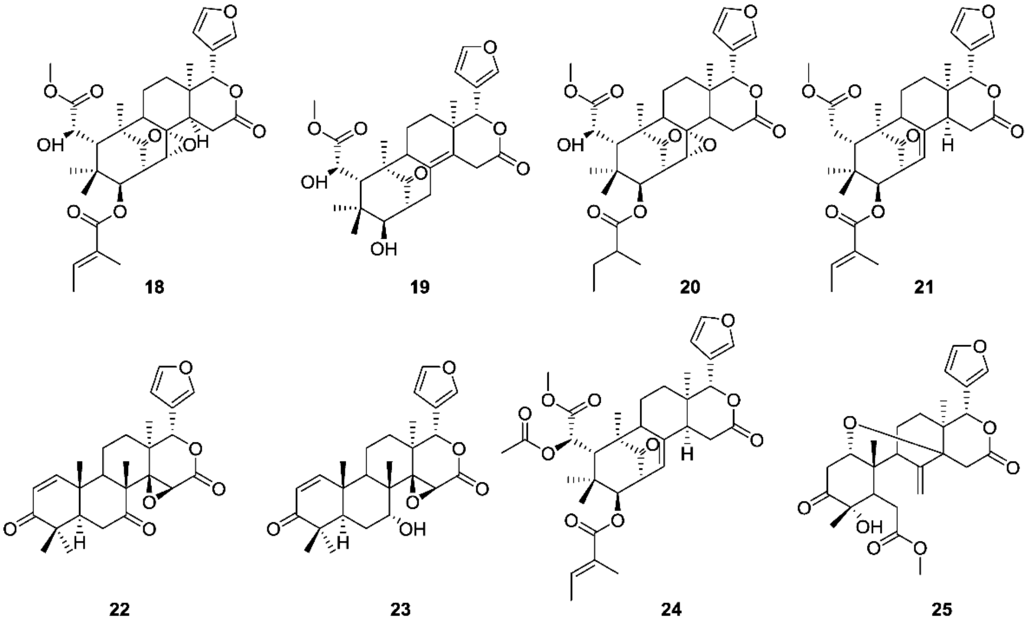

Dataset C: 12 non-peptidic compounds

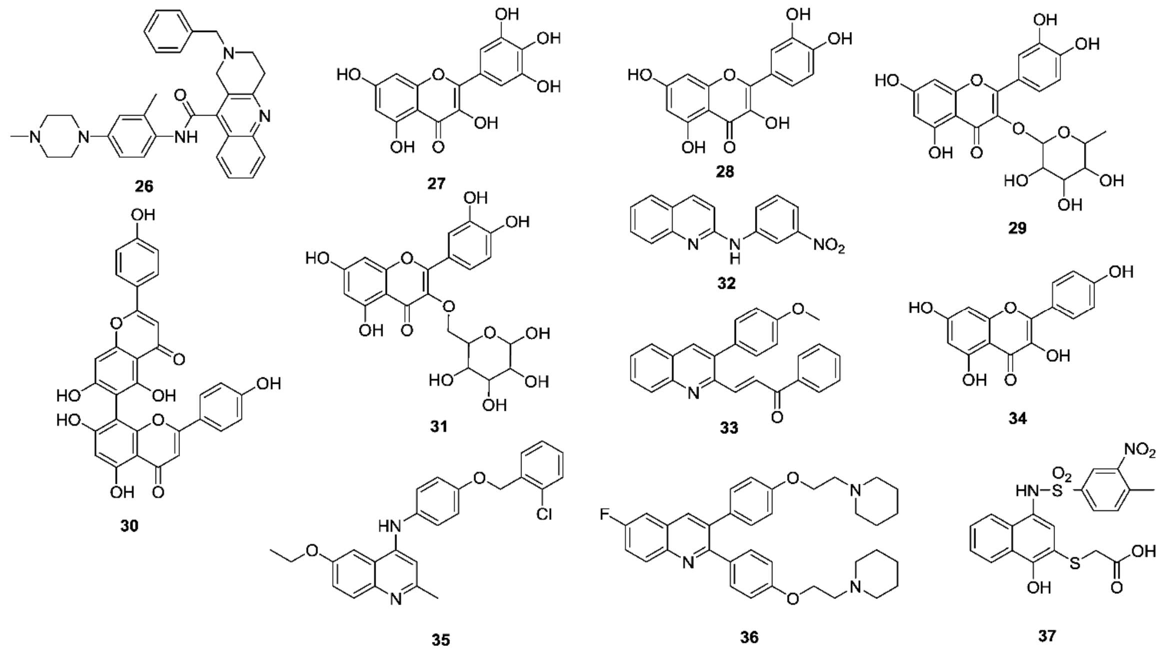

Figure S7. Structure of all anti-dengue virus compounds in Figure 4.
